# Androgen-Modulating Drugs and Severe Complications After ST-Elevation Myocardial Infarction

**DOI:** 10.1001/jamanetworkopen.2026.30774

**Published:** 2026-08-25

**Authors:** Hannah Colldén, Christina E. Lundberg, Lena Björck, Björn Redfors, Annika Rosengren, Åsa Tivesten

**Affiliations:** 1 Wallenberg Laboratory for Cardiovascular and Metabolic Research, Department of Molecular and Clinical Medicine, Institute of Medicine, Sahlgrenska Academy, University of Gothenburg, Sweden; 2 Department of Pharmaceuticals, Sahlgrenska University Hospital, Gothenburg, Region Västra Götaland, Sweden; 3 Department of Food and Nutrition and Sport Science, Faculty of Education, University of Gothenburg, Gothenburg, Sweden; 4 Department of Molecular and Clinical Medicine, Institute of Medicine, Sahlgrenska Academy, University of Gothenburg, Gothenburg, Sweden; 5 Department of Cardiology, Sahlgrenska University Hospital, Region Västra Götaland, Gothenburg, Sweden; 6 Department of Medicine Geriatrics and Emergency Medicine, Sahlgrenska University Hospital, Östra Hospital, Region Västra Götaland, Gothenburg, Sweden; 7 Department of Endocrinology, Sahlgrenska University Hospital, Region Västra Götaland, Gothenburg, Sweden

## Abstract

**Importance:**

Male sex and endogenous testosterone have been shown to exacerbate neutrophilia and cardiac injury in myocardial infarction (MI), raising questions about protective actions of androgen-modulating drugs in acute ST-elevation MI (STEMI).

**Objective:**

To determine whether the use of 5-alpha reductase inhibitors (5αRIs) or androgen deprivation therapy (ADT), drugs that lower androgen actions, is associated with a reduced frequency of severe complications after percutaneous coronary intervention (PCI)–treated STEMI in men.

**Design, Setting, and Participants:**

Nationwide, registry-based cohort study of men aged 35 to 84 years with first-time STEMI treated with PCI from January 2006 to December 2022 in Sweden. Propensity score matching was used to match men treated with 5αRIs or ADT with untreated control patients. The primary outcome was a composite of mortality within 30 days and severe in-hospital complications. Data were analyzed in June 2026.

**Main Outcomes and Measures:**

The primary outcome was a composite of mortality within 30 days and severe in-hospital complications (any of: cardiac arrest and resucitatation, atrioventricular block stage II or III, cardiogenic schock, new-onset atrial fibrillation or flutter, severely depressed left ventricular function, or mechanical complication). Odds ratios were calculated using conditional logistic regression.

**Results:**

In this study of men with PCI-treated STEMI, 1305 men treated with 5αRI (mean [SD] age, 74.2 [6.7] years) were matched to 6463 control patients, and 358 men treated with ADT (mean [SD] age, 76.2 [6.2] years) were matched to 1774 control patients. The odds of experiencing a severe complication were lower in men treated with 5αRIs (odds ratio [OR], 0.80; 95% CI, 0.69-0.93). No significant association was observed for men treated with ADT compared with their matched control patients (OR, 1.10; 95% CI, 0.83-1.45).

**Conclusions and Relevance:**

In this cohort study of men with PCI-treated STEMI, use of 5αRI but not ADT was associated with a lower risk of severe complications. These results warrant further research into the role of androgens and drugs that affect androgen levels in the acute phase of STEMI.

## Key Points

**Question:**

Is treatment with androgen-modulating drugs such as 5-alpha reductase inhibitors (5αRIs) or androgen deprivation therapy associated with reduced frequency of complications after ST-elevation myocardial infarction (STEMI)?

**Findings:**

In this cohort study of 9900 male patients admitted to the hospital with STEMI and treated with percutaneous coronary intervention, the odds of any severe complication were significantly lower in patients treated with 5αRI. No significant difference was seen for patients receiving androgen deprivation therapy.

**Meaning:**

These findings suggest androgen modulation with 5αRIs may influence early clinical outcomes after STEMI and warrants further investigation.

## Teaser

This cohort study evaluates whether the use of 5-α reductase inhibitors or androgen deprivation therapy, drugs that lower androgen actions, is associated with a reduced frequency of severe complications after percutaneous coronary intervention–treated ST-elevation myocardial infarction in men.

## Introduction

Despite substantial advances in recent decades, morbidity and mortality after a myocardial infarction (MI) remain significant.^1^ Larger infarct sizes are linked to adverse outcomes and severe complications, such as mortality, cardiac arrest, cardiogenic shock, and mechanical complications.^2^ Studies indicate that men have larger infarct sizes than women, even in relation to area at risk.^3,4^ Further, when deaths outside hospital are taken into account, men appear to have a worse short-term prognosis after first-time MI.^5^ In a recent translational study, we showed that: (1) neutrophilia and infarct sizes were greater in male vs female mice after experimental MI-reperfusion and in men vs women after reperfusion of first-time ST-elevation MI (STEMI); and (2) higher testosterone levels in males vs females promoted neutrophil egress from bone in acute MI via actions in bone marrow stromal cells.^6^ These findings indicate that androgens increase inflammation in the acute phase of a STEMI.^6^ This inflammation could contribute to cardiac injury in the acute phase, although inflammatory processes are also involved in myocardial healing after MI.^7^

Drugs that decrease systemic androgen levels or actions are frequently prescribed to the older male population. Five-alpha reductase inhibitors (5αRIs), used in benign prostate hyperplasia, decrease local conversion of testosterone to the more potent androgen dihydrotestosterone, and thereby reduce androgen action in tissues.^8^ In Sweden from 2020 to 2024, 10% of men aged 70 to 79 years were prescribed 5αRIs.^9^ In prostate cancer, drugs that decrease the circulating levels or receptor binding of androgens are used as androgen deprivation therapy (ADT) for advanced disease.^10,11,12^ It is unknown whether 5αRIs or ADT are associated with myocardial infarct size or the risk of severe complications after MI.

Based on our findings that testosterone exacerbates neutrophilia and cardiac injury in MI, we hypothesized that men treated with 5αRIs or ADT at the time of a STEMI would experience a reduced inflammatory response, resulting in smaller infarct sizes and fewer early complications. To test this hypothesis, we conducted a registry-based matched cohort study using a nationwide quality register linked to Swedish national registers to evaluate whether ongoing therapy with 5αRIs or ADT at the time of percutaneous coronary intervention (PCI)–treated STEMI was associated with fewer severe complications in men.

## Methods

### Study Cohort

We conducted a hypothesis-driven, registry-based, matched cohort study using the nationwide quality register Swedish Web-system for Enhancement and Development of Evidence-based care in Heart disease Evaluated According to Recommended Therapies (SWEDEHEART), specifically the subregister Swedish Register of Information and Knowledge about Swedish Heart Intensive Care Admissions (RIKS-HIA/SWEDEHEART-ACS). All hospitals in Sweden with acute cardiac care report to this register, with all patients admitted to coronary care units registered.^13^ Patient information from SWEDEHEART was linked with information from the Swedish National Patient Register (contains all hospitalizations and hospital outpatient visits in Sweden), the Cause of Death Register, the Swedish Prescribed Drug Register (contains all prescribed and dispensed medications in Sweden since 2006), and the Longitudinal Integrated Database for Health Insurance (LISA) through the individual personal identity number assigned to all Swedish residents. The study was approved by the regional ethical review board in Gothenburg (DNR: 262-17), which waived the requirement for informed consent because this register-based study used pseudonymized data. The study follows recommendations from the Strengthening the Reporting of Observational Studies in Epidemiology (STROBE) reporting guideline and Reporting of Studies Conducted Using Observational Routinely Collected Health Data (RECORD).^14,15^

From the SWEDEHEART registry, we selected all patients who fulfilled the following criteria: (1) male sex, (2) first registered MI, (3) MI classified as STEMI, (4) treated with PCI, (5) aged 35 to 84 years, and (6) event date between January 2006 and December 2022. Individuals with a reported height below 140 cm, weight below 40 kg, or body mass index (BMI; calculated as weight in kilograms divided by height in meters squared) above 60 were excluded (23 patients). Additional exclusion criteria were treatment with testosterone (Anatomical Therapeutic Chemical [ATC] code G03BA03; 260 patients) or the CYP17a inhibitor abiraterone (ATC code L02BX03; 5 patients), which may have a distinct cardiovascular risk profile not directly related to its antiandrogenic action but to effects on mineralocorticoids and the concomitant administration of prednisolone.^16^

### Exposure

STEMI patients were classified as exposed to 5αRIs, exposed to ADT, or unexposed at the time of their MI. Exposure to 5αRIs or ADT was identified in the prescribed drug register using the following ATC codes: G04CB0 and D11AX10 for 5αRIs; and G03HA01, L02AE, L02BB, and L02BX02 for ADT (eTable 1 in Supplement 1). Patients exposed to both drug classes were classified as ADT-exposed. Exposure required at least 1 dispensed prescription of the drug before the MI, with a dispensed amount (measured as number of tablets for oral formulations and number of daily defined doses for injections) sufficient to cover the MI date. To account for inconsistencies in dosing, adherence, and drug collection, a grace period of 20% of the expected treatment duration was allowed (ie, 100 tablets were assumed to last up to 120 days). To qualify as unexposed, no dispensed prescription of 5αRIs or ADT was allowed in the 12 months before the MI date (ie, individuals with a dispensed prescription of 5αRIs or ADT outside the exposure window but within the 12 months before the MI were excluded). In a sensitivity analysis, patients whose first dispensing occurred 90 days or less before MI were excluded and the cohort was rematched using the same procedure as in the main analysis.

In the 5αRI group, 1267 individuals (97.1%) were prescribed 5-mg finasteride and 38 (2.9%) were prescribed 0.5-mg dutasteride. In the ADT group, 141 individuals (39.4%) were prescribed antiandrogens, 161 (44.7%) were prescribed gonadotropin-releasing hormone (GnRH) agonists, and 56 (15.9%) were prescribed both types. No individuals were prescribed a GnRH antagonist. Thirteen of the individuals in the ADT group were also prescribed a 5αRI.

### Covariates

Information on comorbidities present before admission for STEMI (diabetes, hypertension, previous stroke, angina pectoris, coronary heart disease, heart failure, atrial fibrillation, dementia, chronic obstructive lung disease [COPD], and chronic kidney failure; *International Statistical Classification of Diseases and Related Health Problems, Tenth Revision [ICD-10]* codes in eTable 2 in Supplement 1) was collected from SWEDEHEART and the Swedish National Patient Register (NPR). Data from the Swedish NPR have good diagnostic accuracy for most diagnoses and low missingness.^17^ A diagnosis of diabetes in the registers or a dispensed prescription of diabetes drugs was counted as diabetes. To further account for comorbidity burden we used data from the Drug Register to determine drug usage before the MI. In Sweden, most prescriptions for chronic medications are dispensed for 90 days, and we added an extra 30 days (ie, 120 days before the MI) to account for missed doses and early or late dispensing. We extracted information from the Drug Register on prescriptions for beta blockers, anticoagulants, platelet inhibitors, diuretics, nitrates, calcium antagonists, renin-angiotensin-aldosterone system inhibitors, lipid-lowering drugs, drugs for dementia, antidepressants, and inhalers used in COPD or asthma (ATC codes in eTable 3 in Supplement 1).

BMI was calculated from height and weight registered in SWEDEHEART at the time of admission for STEMI. Information about education level was obtained from the national database LISA that has around 98% coverage for education level.^18^ Postsecondary education was defined as any postsecondary education (college or university studies). Inpatient hospitalizations (defined as hospital admissions with unique dates) during the 2 years preceding the MI were identified from the NPR.

### Outcome

Our primary end point was a composite of death within 30 days after admission for STEMI or any of the following in-hospital events registered in SWEDEHEART in connection with the MI hospitalization: (1) cardiac arrest and resuscitation, (2) atrioventricular (AV) block stage II or III, (3) cardiogenic shock, (4) new-onset atrial fibrillation or flutter, (5) severely depressed left ventricular function (ejection fraction [EF] <30%), or (6) mechanical complications (acute mitral regurgitation caused by papillary muscle rupture, ventricular septal defect, or left ventricular free wall rupture). In SWEDEHEART, left ventricular function is assessed according to routine clinical practice, and the timing of assessment is not standardized across patients. SWEDEHEART records the last EF measurement obtained during the index hospitalization, and this variable has been validated.^19^ In the register, EF is reported in 4 categories: less than 30%, 30% to 39%, 40% to 49%, and 50% or greater.^19^ In our analysis, EF less than 30% was defined as a severe complication. The Swedish Cause of Death Register was used for collecting information on date and cause of death. For individuals who died within 30 days after admission for STEMI, the death was defined as cardiovascular disease-related if the main or contributing cause of death was an *ICD-10* code starting with *I*.

### Statistical Analysis

Data management and statistical analyses were performed in R version 4.4.2 (R Project for Statistical Computing) and finalized in June 2026. Missing data in background variables from SWEDEHEART and from LISA were imputed using multivariate imputation from chained equations with 5 iterations using the R package mice.^20^ To balance covariates between comparison groups, we used propensity score matching based on age, event date, BMI, current smoking status, university education, comorbidities (eTable 2 in Supplement 1), and drug treatment (eTable 3 in Supplement 1). Matching was performed using the MatchIt package in R,^21^ separately for 5αRIs and ADT and performed once per imputed dataset. Propensity scores were estimated using logistic regression and each exposed individual was matched to 5 control patients from the unexposed population using nearest neighbor matching on the logit of the propensity score without replacement, with a caliper of 0.2 × SD. Covariate balance was assessed using standardized mean differences with values less than 0.1 indicating adequate balance.

Presented baseline characteristics and outcomes are from the first imputed dataset. Comparable distributions were observed across all 5 imputations. To account for the matched design, conditional logistic regression models were fitted separately within each of the 5 imputed datasets. Odds ratios (ORs) and standard errors from each imputation were then combined according to Rubin rules to obtain pooled estimates and CIs, incorporating both within- and between-imputation variability. ORs for the individual components of the combined outcome were calculated for the first imputed dataset using the same regression model. In a sensitivity analysis, any inpatient hospitalization was added as a covariate in the main conditional logistic regression model. Results were considered statistically significant if the 95% CI did not cross 1.

## Results

**Figure 1.**
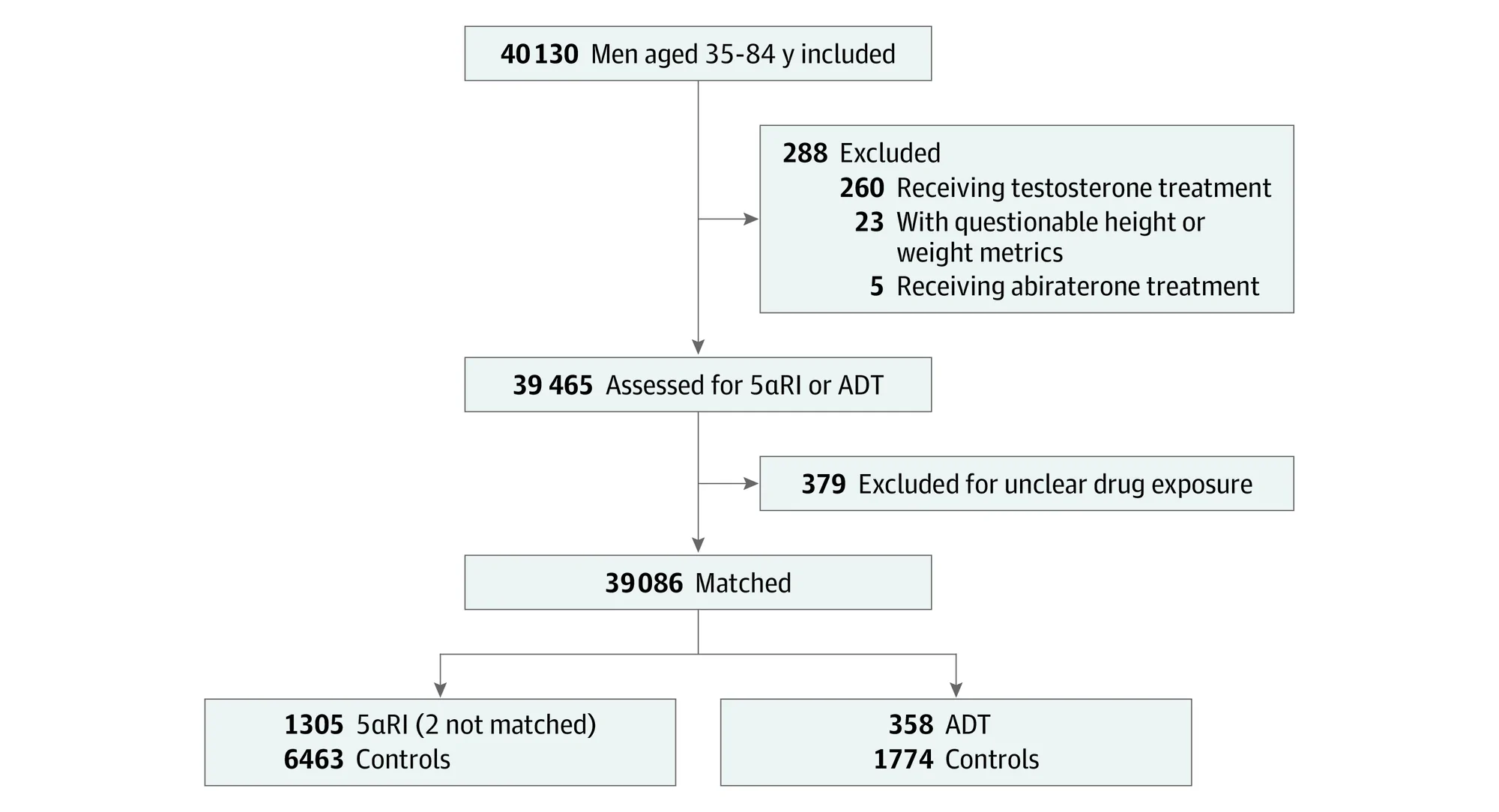
Cohort Selection Flowchart Cohort selection with matching of control patients from the Swedish national quality register SWEDEHEART. 5αRI indicates 5α reductase inhibitor; ADT, androgen deprivation therapy.

In the cohort of 40 130 men with PCI-treated STEMI, 1305 men treated with 5αRIs (mean [SD] age 74.2 [6.7] years) and 358 men treated with ADT (mean [SD] age 76.2 [6.2] years) were matched to 6463 and 1774 control patients, respectively. (Figure 1).

As expected, men treated with 5αRIs or ADT were considerably older and had more comorbidities and other drug treatments than the control patients without such treatment (eTable 4 in Supplement 1). We therefore used a propensity score–matched analysis and selected 5 matched control patients per exposed individual for each of the 2 treatment groups. Using this matching approach, the examined baseline variables were well balanced between men treated with 5αRIs or ADT and their respective control patients (Table 1).

**Table 1.** Characteristics of the Matched Cohorts of Men With Percutaneous Coronary Intervention–Treated ST-Elevation Myocardial Infarction

Characteristic	Participants, No. (%)		SMD	Participants, No. (%)		SMD
	5αRI (n = 1305)	Control (n = 6463)		ADT (n = 358)	Control (n = 1774)	
Age, mean (SD), y	74.15 (6.68)	74.18 (6.80)	<0.01	76.18 (6.23)	76.12 (6.24)	<0.01
Event date, mean (SD)	June 28, 2016 (4.15 y)	May 16, 2016 (4.17 y)	0.03	December 23, 2015 (4.16 y)	December 1, 2015 (4.21 y)	0.01
BMI, mean (SD)^a^	26.57 (3.66)	26.57 (3.75)	<0.01	26.50 (3.91)	26.56 (3.76)	0.02
Current smoker^a^	559 (42.8)	2755 (42.6)	<0.01	158 (44.1)	795 (44.8)	0.01
Postsecondary education^a^	307 (23.5)	1502 (23.2)	<0.01	58 (16.2)	300 (16.9)	0.02
Comorbidities						
Diabetes	257 (19.7)	1205 (18.6)	0.03	74 (20.7)	357 (20.1)	0.01
Hypertension	806 (61.8)	3936 (60.9)	0.02	204 (57.0)	992 (55.9)	0.02
Angina	175 (13.4)	802 (12.4)	0.03	50 (14.0)	227 (12.8)	0.03
Ishemic heart disease	125 (9.6)	588 (9.1)	0.02	31 (8.7)	145 (8.2)	0.02
Heart failure	46 (3.5)	228 (3.5)	<0.01	15 (4.2)	69 (3.9)	0.02
Previous stroke	117 (9.0)	510 (7.9)	0.04	35 (9.8)	172 (9.7)	<0.01
Atrial fibrillation	125 (9.6)	588 (9.1)	0.02	31 (8.7)	145 (8.2)	0.02
Dementia	41 (3.1)	202 (3.1)	<0.01	10 (2.8)	49 (2.8)	<0.01
Chronic kidney disease	51 (3.9)	218 (3.4)	0.03	10 (2.8)	46 (2.6)	0.01
COPD or asthma	117 (9.0)	547 (8.5)	0.02	45 (12.6)	211 (11.9)	0.02
Drugs before myocardial infarction						
Anticoagulants	115 (8.8)	525 (8.1)	0.03	24 (6.7)	106 (6.0)	0.03
Platelet inhibitors	258 (19.8)	1263 (19.5)	<0.01	77 (21.5)	378 (21.3)	<0.01
COPD or asthma inhalers	132 (10.1)	587 (9.1)	0.04	42 (11.7)	215 (12.1)	0.01
Antidepressants	115 (8.8)	529 (8.2)	0.02	30 (8.4)	147 (8.3)	<0.01
Dementia medication	37 (2.8)	186 (2.9)	<0.01	8 (2.2)	38 (2.1)	<0.01
Parkinson medication	23 (1.8)	118 (1.8)	<0.01	4 (1.1)	19 (1.1)	<0.01
Lipid lowering drugs	313 (24.0)	1468 (22.7)	0.03	77 (21.5)	384 (21.6)	<0.01
Antihypertensives	23 (1.8)	97 (1.5)	0.02	1 (0.3)	5 (0.3)	<0.01
Diuretics	187 (14.3)	871 (13.5)	0.03	63 (17.6)	305 (17.2)	0.01
RAAS-inhibitors	509 (39.0)	2485 (38.4)	0.01	120 (33.5)	578 (32.6)	0.02
Betablockers	343 (26.3)	1643 (25.4)	0.02	72 (20.1)	371 (20.9)	0.02
Calcium antagonists	363 (27.8)	1753 (27.1)	0.02	88 (24.6)	446 (25.1)	0.01
Nitrates	90 (6.9)	401 (6.2)	0.03	33 (9.2)	153 (8.6)	0.02

Abbreviations: 5αRI, 5-alpha reductase inhibitor; ADT, androgen deprivation therapy; BMI, body mass index (calculated as weight in kilograms divided by height in meters squared); COPD, chronic obstructive pulmonary disease; RAAS, renin-angiotensin-aldosterone system; SMD, standard mean difference.

^a^ Multiple imputation by chained equations for missing data in smoking (4.2% missing), BMI (6.8% missing), and education (1.4% missing) were performed 5 times with comparable baseline variables; imputation 1 is shown.

The frequency of the components of the combined outcome are listed in Table 2. The absolute risk of a severe complication in the patients treated with 5αRI was 20.8% (271 patients) while the corresponding risk for matched control patients was 24.3% (1568 control patients), corresponding to an absolute risk difference of 3.5 percentage points (Table 2). Among the patients who died within 30 days after MI, cause of death was cardiovascular-related in 82 patients (93.2%) treated with 5αRI, 408 control patients (90.1%) matched to the 5αRI group, 29 patients (93.2%) treated with ADT, and 124 control patients (96.9%) treated with ADT (imputation 1, with comparable results for the other imputations).

**Table 2.** Components of the Composite Outcome (Severe Myocardial Infarction [MI] Complications) Reported Descriptively^a^

Component	Participants, No. (% of group)			
	5αRI (n = 1305)	Control (n = 6463)	ADT (n = 358)	Control (n = 1774)
30 d Mortality	88 (6.7)	453 (7.0)	31 (8.7)	128 (7.2)
In-hospital cardiac arrest and resuscitation	79 (6.1)	464 (7.2)	22 (6.1)	111 (6.3)
Cardiogenic chock	53 (4.1)	336 (5.2)	19 (5.3)	76 (4.3)
Mechanical complication	6 (0.5)	25 (0.4)	0	9 (0.5)
AV block II or III	29 (2.2)	194 (3.0)	11 (3.1)	55 (3.1)
Severe left ventricular dysfunction	73 (6.1)	451 (7.7)	26 (8.1)	144 (9.1)
New fibrillation or flutter	95 (7.3)	580 (9.0)	32 (8.9)	165 (9.3)
Severe MI combined outcome	271 (20.8)	1568 (24.3)	101 (28.2)	439 (24.7)

Abbreviations: 5αRI, 5-alpha reductase inhibitor; ADT, androgen deprivation therapy; AV, atrioventricular.

^a^ Imputation 1 out of 5 is shown, results were similar across imputations.

**Figure 2.**
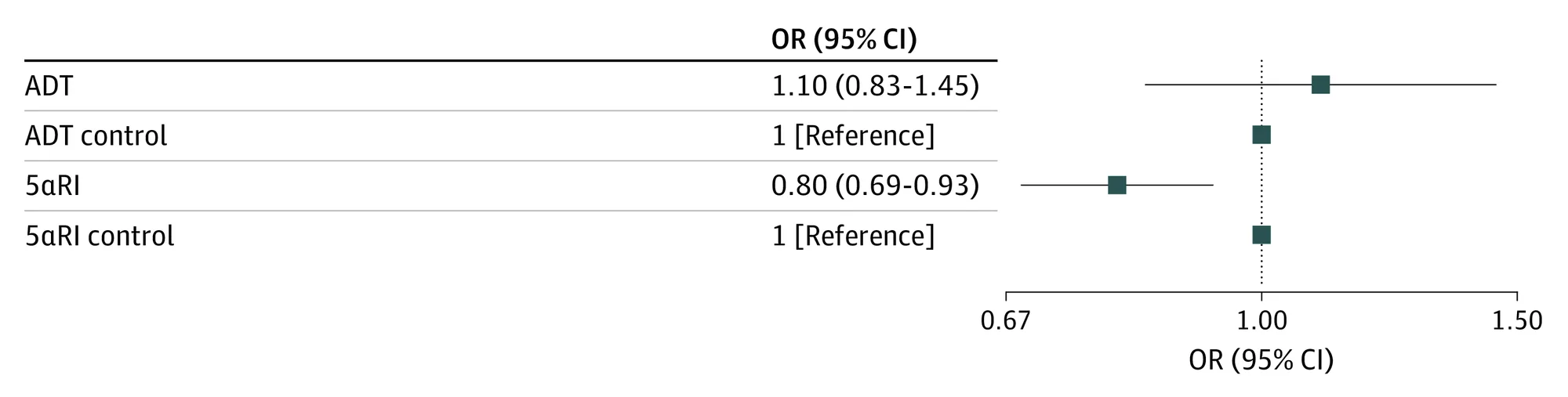
Dot Plot Showing Severe Complications After Percutaneous Coronary Intervention–Treated ST-Elevation Myocardial Infarction Pooled ORs with 95% CIs for severe complications of percutaneous coronary intervention–treated ST-elevation myocardial infarction (composite outcome) for men treated with 5αRIs or ADT, each compared with matched control patients, shown on a logarithmic scale. Estimates were derived from conditional logistic regression models. Analyses were performed across 5 multiply imputed and matched datasets with results combined using Rubin rules. 5αRI indicates 5α reductase inhibitor; ADT, androgen deprivation therapy; OR, odds ratio.

In the propensity score–matched analysis, the odds of severe complications after MI were lower in patients treated with 5αRI (pooled OR, 0.80; 95% CI, 0.69-0.93) but not in patients treated with ADT (OR, 1.10; 95% CI, 0.83-1.45) compared with their respective matched control patients (Figure 2). ORs for the individual components of the combined outcome were broadly consistent across components (eTable 5 in Supplement 1).

Excluding patients with 90 or fewer days’ exposure for 5αRI or ADT yielded similar results (5αRI: 1191 patients; pooled OR, 0.76; 95% CI, 0.65-0.89; ADT: 323 patients; pooled OR, 1.10; 95% CI, 0.80-1.50). Health care utilization was accounted for in a sensitivity analysis by additional adjustment for any inpatient hospitalization during the 2 years preceding the MI (eTable 6 in Supplement 1). Results were materially unchanged after this additional adjustment (5αRI: pooled OR, 0.80; 95% CI, 0.68-0.93; ADT: pooled OR, 1.07; 95% CI, 0.81-1.42).

## Discussion

In this observational study based on Swedish registers, we investigated whether ongoing treatment with 5αRIs or ADT is associated with severe complications after PCI-treated STEMI in men. We observed that the odds of severe complications after an MI were lower in patients treated with 5αRIs at the time of PCI-treated STEMI compared with propensity-score matched control patients. For patients treated with ADT, however, no corresponding association was found.

This is the first study that addresses a potential association between the use of drugs that lower androgen action and the occurrence of severe acute complications following a STEMI. Our finding that patients treated with a 5αRI at the time of PCI-treated STEMI experienced fewer severe complications post MI is in accordance with our hypothesis, based on our earlier translational study showing that male sex and testosterone increase the neutrophilic response, cardiac injury, and mortality in acute MI.^6^ Evidence in support of actions in the immune system mediating adverse cardiovascular effects of testosterone is provided by the recent randomized clinical trial Testosterone Replacement Therapy for Assessment of Long-term Vascular Events and Efficacy Response in Hypogonadal Men (TRAVERSE),^22^ which showed that a testosterone-induced increase in neutrophil counts was associated with increased risk of thromboembolic events in men. 5αRIs block the conversion of testosterone to the more potent androgen dihydrotestosterone in target tissues, thereby reducing local androgen receptor activation.^23^ Although local dihydrotestosterone production is most prominent in classical androgen-responsive tissues such as the prostate, skin, and hair follicles,^23^ 5α-reductase is expressed in other tissues and cell types, including bone marrow stromal or mesenchymal cells.^24^ Thus, speculatively, 5αRI use may mimic our mouse model of androgen receptor depletion in bone marrow stromal cells, which—similar to castration—results in reduced neutrophilia and a smaller cardiac injury after MI.^6^ This registry study could not determine whether 5αRI exposure was associated with less neutrophilia as we lacked data on inflammatory markers. However, in support of a potential effect of 5αRIs on bone marrow neutrophil egress in males, pretreatment with the 5αRI finasteride has been shown to reduce tissue infiltration of neutrophils in an experimental trauma-hemorrhage model.^25^

In contrast to 5αRI treatment, ADT treatment was not associated with a lower risk of severe complications after PCI-treated STEMI. Important differences between the 5αRI and ADT analyses should be noted. Although both 5αRIs and ADT are prescribed to older men, 5αRIs are used to treat a benign condition (prostate hyperplasia) whereas ADT is prescribed to patients with advanced prostate cancer, potentially introducing selection bias, such as access to PCI or via differences in health care–seeking behavior. The treatments also differ pharmacodynamically: 5αRIs reduce the production of dihydrotestosterone from testosterone in target tissues, whereas ADT drugs have heterogeneous mechanisms for decreased androgen action through decrease in circulating testosterone or inhibition of androgen receptor binding.^26^ This heterogeneity may increase variability in the ADT group. Furthermore, the ADT group was substantially smaller, resulting in relatively lower power than the 5αRI analysis.

The cardiovascular actions of androgens may be beneficial or detrimental, depending on setting, target tissue, type of cardiovascular disease, and disease stage.^27^ This may be due to different mechanisms at play, including (anti)atherogenic,^28^ metabolic, cardiotropic,^29^ and immunomodulatory^6^ actions of androgens. While the present study addresses a specific question regarding adverse effects of androgens in acute PCI-treated STEMI, the cardioprotective actions of androgens in men are illustrated by the increased cardiovascular risk associated with ADT, potentially mediated by adverse effects of ADT on obesity, hypertension, and metabolic risk factors.^30,31^ In contrast, the cardiovascular and metabolic effects of 5αRIs have not been as thoroughly studied or characterized.^8^ Our present data contribute to a growing body of evidence supporting a detrimental role of androgens in the acute phase of a STEMI.^6^ Further studies are required to confirm our findings in other datasets.

### Limitations and Strengths

Limitations of the current study include the lack of direct measurements of infarct size and inflammation and the use of severe complications as a proxy for larger cardiac injury. Troponin measurements are recorded in SWEDEHEART, but since the timing of the troponin measurements are not directed at detecting the maximum value, these measurements do not accurately reflect infarct size in a PCI-treated STEMI population and were not used. Details on door-to-balloon time, total ischemic time, and Killip class, which are strong predictors of prognosis following STEMI, were not available in our dataset, and we cannot exclude that these differ between the exposure groups.

The patients treated with ADT differ from their matched control patients in that they have advanced cancer, and this may be linked to systemic inflammation and other oncologic treatment that may not be captured by the available covariates and may influence the short-term outcomes after PCI-treated STEMI. Also, ADT has other, long-term, negative effects on the heart,^32^ possibly obscuring any protective effect in the acute MI situation. With a larger dataset and data on prostate cancer stage, treatment, and duration, investigation of subgroups within ADT and prostate cancer may provide additional information. As with all observational studies, we cannot infer causality from observed associations and there may be residual confounding between patients treated with 5αRI or ADT and control patients that our propensity score matching was not able to capture.

Strengths of this study include the use of the nationwide quality register SWEDEHEART, which includes more than 90% of patients in Sweden hospitalized with STEMI and treated with PCI.^33^ This minimizes selection bias and provides a large pool of control patients, enabling propensity score matching to attain groups with similar baseline characteristics. SWEDEHEART has been validated as a reliable platform for registry-based research.^34^ The study period reflects both the availability of the Swedish Drug Register and contemporary STEMI care; with only minor improvements in PCI and mortality rates in Sweden during this period.^33^ Linkage to Swedish national registers provides accurate information on comorbidities and drugs dispensed at pharmacies, eliminating the risk of recall bias. Inclusion of BMI and smoking in the propensity score matching further strengthens this study. Including all-cause mortality in the composite outcome avoids competing risks bias.

## Conclusions

In this cohort study of 40 130 men with PCI-treated STEMI, 1305 men treated with 5αRI and 358 men treated with ADT were compared with up to 5 propensity score–matched control patients per exposed individual using linked nationwide Swedish quality and health registers. We tested the hypothesis that men treated with drugs that lower androgen action at the time of a PCI-treated STEMI would experience fewer early complications and found decreased odds of severe complications with 5αRI treatment but not with ADT at the time of STEMI. These results warrant further research into the role of androgens and drugs that affect androgen levels in the acute phase of STEMI.

## Notes

Supplement 1. eTable 1. ATC Codes (and Examples of Substances) Used to Identify Exposure to 5αRIs/ADT eTable 2. ICD Codes for Comorbidities From the National Patient Register eTable 3. ATC Codes for Drug Treatment From the Prescribed Drug Register eTable 4. Baseline Variables for the Full Cohort of Men 35 to 84 Years in Sweden With a First-Time ST-Elevation Myocardial Infarction With Percutaneous Coronay Intervention (PCI) 2006-2022 Before Imputation and Matching eTable 5. Odds Ratios (OR) With 95% CI for Severe Complications of ST-Elevation Myocardial Infarction Reported Descriptively for the Components of the Composite Outcome eTable 6. Number of Hospitalizations 2 Years Before the PCI-Treated STEMI

Supplement 2. Data Sharing Statement
