# Supplementary material for: Androgen-Modulating Drugs and Severe Complications After ST-Elevation Myocardial Infarction
Source: JAMA Netw Open. 2026 Aug 25;9(8):e2630774. doi: 10.1001/jamanetworkopen.2026.30774 (PMC13507922; doi:10.1001/jamanetworkopen.2026.30774)
Supplement: Supplementary file 1 [file jamanetwopen-e2630774-s001.pdf]

Supplemental Online Content

Colldén H, Lundberg CE, Björck L, Redfors B, Rosengren A, Tivesten Å. Androgen-modulating drugs and severe complications after ST-elevation myocardial infarction. *JAMA Netw Open*. 2026;9(8):e2630774. doi:10.1001/jamanetworkopen.2026.30774

eTable 1 ATC Codes (and Examples of Substances) Used to Identify Exposure to 5αRIs/ADT

eTable 2 ICD Codes for Comorbidities From the National Patient Register

eTable 3 ATC Codes for Drug Treatment From the Prescribed Drug Register

eTable 4 Baseline Variables for the Full Cohort of Men 35 to 84 Years in Sweden With a First-Time ST-Elevation Myocardial Infarction With Percutaneous Coronay Intervention (PCI) 2006-2022 Before Imputation and Matching

eTable 5 Odds Ratios (OR) With 95% CI for Severe Complications of ST-Elevation Myocardial Infarction Reported Descriptively for the Components of the Composite Outcome

eTable 6 Number of Hospitalizations 2 Years Before the PCI-Treated STEMI

This supplemental material has been provided by the authors to give readers additional information about their work.

eTable 1 ATC Codes (and Examples of Substances) Used to Identify Exposure to 5αRIs/ADT

ATC-code	Name (examples of substances)
5αRI	
G04CB01/2	5-α reductase inhibitors (finasteride, dutasteride), prostate indication
D11AX10	Finasteride, dermatological indication
ADT	
G03HA01	Antiandrogen (cyproterone)
L02AE	GnRH agonists (buserelin, leuprolein, gosereline, triptoreline)
L02BB	Antiandrogens (flutamide, enzalutamide, bicalutamide, apalutamide, darolutamide)
L02BX02	GnRH antagonist (degarelix)

5αRI = five alpha reductase inhibitor, ADT = androgen deprivation therapy, GnRH = Gonadotropin releasing hormone

eTable 2 ICD Codes for Comorbidities From the National Patient Register

ICD-10 code	ICD-9 code	Comorbidity
E10-E11	250	Diabetes (type 1 or 2)
I10- I15	401-405	Hypertension
E78	272	Hyperlipidaemia
I20	410	Angina Pectoris
I25	414	Ischemic Heart Disease
I48	427D	Atrial Fibrillation/Flutter
I50	428	Heart Failure
I61-I64	431-436	Stroke
F00-F07, G30-G31	290, 294, 331	Dementia
N18-N19, N25, N28	585	Chronic Kidney Disease
J44-46	491, 493, 496	COPD or asthma

ICD = International Classification of Diseases, COPD = chronic obstructive lung disease

© 2026 Colldén H et al. *JAMA Network Open*.

eTable 3 ATC Codes for Drug Treatment From the Prescribed Drug Register

ATC code starting with	Drug Group
A10	Diabetes drugs
B01AA, B01AB, B01AF, B01AX	Anticoagulants
B01AC, B01AE	Platelet inhibitors
R03	Inhalers for COPD/asthma
N06A	Antidepressants
N06D	Dementia medication
N04	Parkinson's medication
C02	Antihypertensives
C03	Diuretics
C07	Betablockers
C08	Calcium antagonists
C09	RAAS inhibitors
C10	Lipid lowering drugs

ATC = Anatomical Therapeutical Classification, COPD = chronic obstructive lung disease, RAAS = Renin Angiotensin Aldosterone System

© 2026 Colldén H et al. *JAMA Network Open*.

eTable 4 Baseline Variables for the Full Cohort of Men 35 to 84 Years in Sweden With a First-Time ST-Elevation Myocardial Infarction With Percutaneous Coronay Intervention (PCI) 2006-2022 Before Imputation and Matching

	**5αRI**	**ADT**	**Control**	**SMD**	**Missing (%)**
n	1307	358	37798		
Age (mean (SD))	74.17 (6.68)	76.18 (6.23)	64.00 (10.44)	0.962	0.0
Event date (mean (SD years))	2016-07-01 (4.1)	2015-12-23 (4.2)	2015-07-14 (4.2)	0.154	0.0
BMI (mean (SD))	26.57 (3.65)	26.51 (3.92)	27.28 (4.07)	0.132	6.8
Current smoker (%)	523 (43.2)	149 (44.5)	12079 (33.3)	0.154	4.2
Post-secondary education (%)	304 (23.6)	57 (16.1)	8539 (22.9)	0.125	1.4
Comorbidities (%)					
Diabetes	257 (19.7)	74 (20.7)	5401 (14.3)	0.112	0.0
Hypertension	808 (61.8)	204 (57.0)	15859 (42.0)	0.269	0.0
Angina	175 (13.4)	50 (14.0)	3049 (8.1)	0.126	0.0
Ishemic heart disease	126 (9.6)	31 (8.7)	1554 (4.1)	0.147	0.0
Heart failure	46 (3.5)	15 (4.2)	674 (1.8)	0.095	0.0
Previous stroke	118 (9.0)	35 (9.8)	1533 (4.1)	0.152	0.0
Atrial fibrillation	126 (9.6)	31 (8.7)	1554 (4.1)	0.147	0.0
Dementia	42 (3.2)	10 (2.8)	735 (1.9)	0.054	0.0
Chronic kidney disease	51 (3.9)	10 (2.8)	679 (1.8)	0.085	0.0
COPD or asthma	118 (9.0)	45 (12.6)	1943 (5.1)	0.177	0.0
Drugs before myocardial infarction (%)					
Anticoagulants	116 (8.9)	24 (6.7)	1238 (3.3)	0.158	0.0
Platelet inhibitors	259 (19.8)	77 (21.5)	3630 (9.6)	0.222	0.0
COPD/asthma inhalers	132 (10.1)	42 (11.7)	2230 (5.9)	0.138	0.0
Antidepressants	117 (9.0)	30 (8.4)	2292 (6.1)	0.073	0.0
Dementia medication	38 (2.9)	8 (2.2)	497 (1.3)	0.074	0.0
Parkinson medication	24 (1.8)	4 (1.1)	332 (0.9)	0.056	0.0
Lipid lowering drugs	315 (24.1)	77 (21.5)	4950 (13.1)	0.190	0.0
Antihypertensives	23 (1.8)	1 (0.3)	269 (0.7)	0.101	0.0
Diuretics	188 (14.4)	63 (17.6)	2822 (7.5)	0.207	0.0
RAAS-inhibitors	511 (39.1)	120 (33.5)	9178 (24.3)	0.215	0.0
Betablockers	344 (26.3)	72 (20.1)	5345 (14.1)	0.204	0.0
Calcium antagonists	364 (27.9)	88 (24.6)	5852 (15.5)	0.202	0.0
Nitrates	90 (6.9)	33 (9.2)	1139 (3.0)	0.175	0.0

BMI = Body Mass Index, COPD = chronic obstructive lung disease, RAAS = Renin Angiotensin Aldosterone System, 5αRI = five alpha reductase inhibitor, ADT = androgen deprivation therapy, SMD = Standard Mean Difference

© 2026 Colldén H et al. *JAMA Network Open*.

eTable 5 Odds Ratios (OR) With 95% CI for Severe Complications of ST-Elevation Myocardial Infarction Reported Descriptively for the Components of the Composite Outcome

**OR (95% CI)**	**5αRI**	**ADT**
30-day mortality	0.93 (0.74 - 1.19)	1.22 (0.81-1.84)
In-hospital cardiac arrest and resuscitation	0.82 (0.64 - 1.05)	0.99 (0.61-1.58)
Cardiogenic schock	0.76 (0.56 - 1.02)	1.25 (0.75-2.09)
Mechanical complication	1.16 (0.47 - 2.82)	NA
AV block II or III	0.73 (0.49 - 1.09)	0.97 (0.50 - 1.87)
Severe left ventricular dysfunction	0.76 (0.59 - 0.99)	0.91 (0.59-1.42)
New fibrillation or flutter	0.79 (0.63 - 1.00)	0.93 (0.65-1.43)

NA = not applicable, cannot be calculated as one of the groups had 0 events, 5αRI = five alpha reductase inhibitor, ADT = androgen deprivation therapy, AV = atrioventricular

eTable 6 Number of Hospitalizations 2 Years Before the PCI-Treated STEMI

	**5αRI**	**Control**	**ADT**	**Control**
Median (1-99 percentile)	0 (0-5.0)	0 (0-6.0)	0 (0-5.4)	0 (0-5.3)
Any (%)	24.75	22.50	35.75	23.34

5αRI = five alpha reductase inhibitor, ADT = androgen deprivation therapy, PCI = percutaneous coronary intervention, STEMI = ST-elevation myocardial infarction

© 2026 Colldén H et al. *JAMA Network Open*.
