# Supplementary material for: Androgen-Modulating Drugs and Severe Complications After ST-Elevation Myocardial Infarction
Source: JAMA Netw Open. 2026 Aug 25;9(8):e2630774. doi: 10.1001/jamanetworkopen.2026.30774 (PMC13507922; doi:10.1001/jamanetworkopen.2026.30774)
Supplement: Supplementary file 2 [file jamanetwopen-e2630774-s002.pdf]

Data Sharing Statement

Colldén. Androgen-Modulating Drugs and Severe Complications After ST-Elevation Myocardial Infarction. *JAMA Netw Open*. Published August 25, 2026.
doi:10.1001/jamanetworkopen.2026.30774

Data
Data available: No

Additional Information
Explanation for why data not available: Due to Swedish privacy laws, data are only available from the sources stated in the paper on request to the data providers, fulfilling legal and regulatory requirements and with permission from the Swedish Ethical Review Authority.
